# Long-term follow-up study of low-weight avoidant/restrictive food intake disorder and childhood-onset anorexia nervosa: comparison of autistic eating behaviours

**DOI:** 10.1186/s40337-026-01697-5

**Published:** 2026-07-08

**Authors:** C. R. André Lange, Emma Claesdotter-Knutsson, Peik Gustafsson, Ulf Wallin

**Affiliations:** 1 Child and Adolescent Psychiatry, Department of Clinical Sciences Lund, Medical Faculty, Lund University, Lund, Sweden; 2 Centre of Eating Disorders, Psychiatry Skåne, Lund, Sweden

**Keywords:** Anorexia nervosa, Avoidant/restrictive food intake disorder, Autism, Feeding and eating disorders of childhood, Follow-up studies

## Abstract

**Background:**

Anorexia nervosa (AN) is characterized by restrictive eating associated with weight and shape concerns, whereas avoidant/restrictive food intake disorder (ARFID) is not. Evidence supports an association between AN and autism, and emerging data suggest a similar overlap between ARFID and autism. However, long-term data on autistic eating behaviours in these groups remain scarce. This study examined autistic eating behaviours at long-term follow-up in former patients with childhood-onset AN and low-weight ARFID.

**Methods:**

Participants were 56 former patients treated in specialist child and adolescent eating disorder services in Sweden (37 with AN and 19 with ARFID at treatment start) who were assessed at a mean follow-up of 15.9 years. Autistic eating behaviours were measured with the Swedish Eating Assessment for Autism Spectrum Disorders questionnaire (SWEAA; primary measure). Secondary measures were the Morgan-Russell Outcome Assessment Schedule (M-R OAS) and the Eating Disorder Examination Questionnaire (EDE-Q) [21, 27]. Follow-up assessment also included a structured diagnostic interview (SCID) and measured height and weight. ARFID diagnoses were assigned retrospectively using all available clinical information. Mann-Whitney U tests, Kruskal-Wallis tests, descriptive statistics, and linear regression were used for group comparisons and association analyses.

**Results:**

Both the AN and ARFID groups showed elevated SWEAA scores at follow-up, with no significant difference between groups. Participants with an ongoing eating disorder at follow-up had the highest SWEAA scores, whereas those with no diagnosis had the lowest scores. In both diagnostic groups, the body mass index (BMI) < 20 category showed the lowest SWEAA scores. In the ARFID group, higher BMI was positively associated with SWEAA total score. M-R OAS scores differentiated follow-up diagnostic groups, whereas EDE-Q scores showed no clear pattern across groups.

**Conclusions:**

Autistic eating behaviours were common at long-term follow-up in both childhood-onset AN and low-weight ARFID. Higher SWEAA scores were associated with concurrent psychopathology, particularly an ongoing eating disorder, but autistic eating behaviours were not more pronounced in AN than in ARFID. The unexpectedly low SWEAA scores in the low-BMI group warrant further investigation.

## Abstract (plain language summary)

The long-term outcomes of childhood-onset anorexia nervosa (AN) and avoidant/restrictive food intake disorder (ARFID) are still not fully understood. Research has shown an overlap between autism and AN, and newer studies suggest that ARFID may also be linked to autism-related eating characteristics. In this study, we followed up a group of former patients about 16 years after treatment for AN or ARFID. Participants completed the Swedish Eating Assessment for Autism Spectrum Disorders (SWEAA), an interview designed to establish whether people had mental health conditions, and other standard eating-disorder measures, and their height and weight were recorded. We found that autistic eating behaviours were common in both the AN and ARFID groups at follow-up. People with an ongoing eating disorder had the highest SWEAA scores, whereas those without a psychiatric diagnosis had the lowest scores. Unexpectedly, participants in the low-BMI group had the lowest SWEAA scores in both diagnostic groups. These findings suggest that autistic eating behaviours may remain clinically relevant in long-term follow-up of both AN and ARFID, but that their relationship with weight status is more complex than expected.

## Background

Anorexia nervosa (AN) is a severe eating disorder characterized by restrictive eating, low weight, and overvaluation of body shape and weight, with substantial risks of chronicity and mortality [2, 17]. Avoidant/restrictive food intake disorder (ARFID) also involves restrictive eating and may lead to markedly low weight, but it is not driven by body image disturbance or a thin ideal; instead, food restriction in ARFID is motivated by sensory-based avoidance, lack of interest in food and eating, or concern about aversive consequences of eating [2]. Long-term outcome in severe low-weight ARFID may in some cases be comparable to that seen in AN [18].

There is growing evidence of an association between AN and autism [4, 11]. Proposed mechanisms include rigidity, detail-focused processing, sensory sensitivities, social-communication differences, and difficulties in emotion recognition and flexibility [1, 16, 32]. Clinical and epidemiological studies have reported elevated rates of autism in AN [20, 22, 28]. Eating disturbances in autism include rumination, polydipsia, food neophobia and selective eating. Underlying mechanisms for the eating behaviours in autism include sensory abnormalities, restricted interests, routines, social interaction skills, motor functioning and gastro-intestinal problems [24]. Previous work using questionnaires suggested that autistic eating behaviours are common in AN and may persist after weight gain [13]. Thus, the overlap appears to extend beyond acute starvation effects alone.

There are also reasons to suspect an association between ARFID and autism. ARFID commonly involves sensory-based food avoidance, rigidity, narrow food preferences, and early-onset feeding problems, all of which overlap clinically with presentations frequently seen in autistic individuals [5]. Emerging empirical findings support this link: in a Swedish twin cohort, 13.8% of individuals meeting ARFID-phenotype criteria had co-occurring autism [31], and a recent meta-analysis reported autism in 16.3% of patients with ARFID [25].

Despite increasing evidence of overlap between restrictive eating disorders and autism, long-term follow-up studies remain scarce, particularly in ARFID. A better understanding of autistic eating behaviours in the long term may improve clinical follow-up, help identify patients with persistent psychiatric burden, and refine conceptual models of overlap between eating disorders and autism. The present study therefore compared autistic eating behaviours at long-term follow-up in former patients with childhood-onset AN and low-weight ARFID. We examined whether autistic eating behaviours differed between AN and ARFID and whether they varied according to follow-up diagnostic status and category.

## Methods

### Participants and setting

**Fig. 1:**
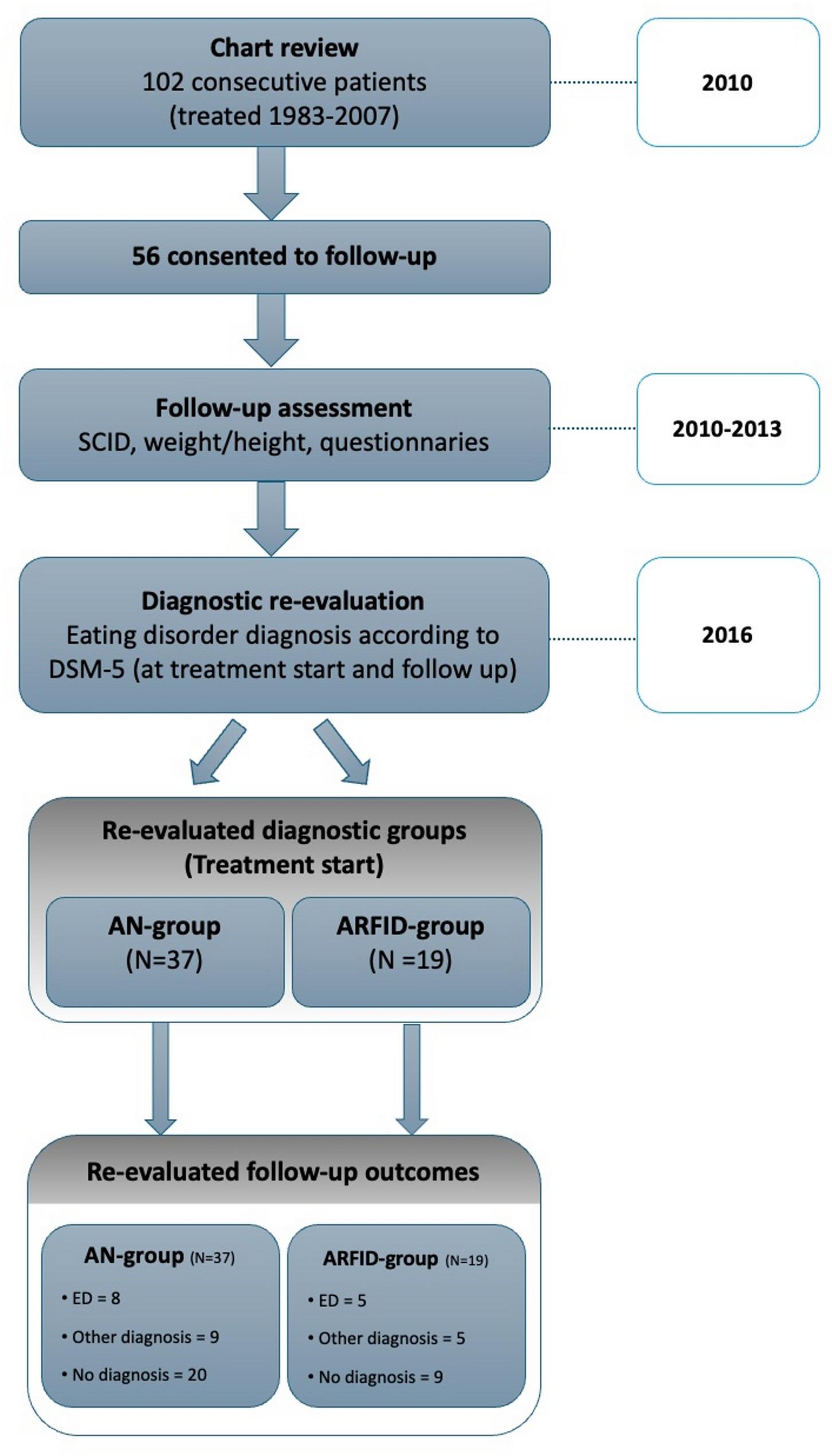
Participants and procedure

The study was based on the same 56 participants (53 women and 3 men) included in a previous long-term follow-up study of early-onset AN and low-weight ARFID [18] (Fig. 1). Participants were former patients from specialist child and adolescent eating disorder services in Sweden. Of the 56 participants, 37 had AN and 19 had ARFID diagnosis at treatment start. Follow-up occurred at a median of 15.9 years after treatment initiation (range 7–29 years). At follow-up, 29 participants had no current psychiatric diagnosis, 14 had a psychiatric diagnosis other than an eating disorder, and 13 met criteria for an eating disorder. The study was approved by the regional ethical review board at Lund University (DNr. 2009/619; on 10 February 2010). Written informed consent was obtained from all study participants.

From the original treated cohort, 45% did not participate in the follow-up. Participants and non-participants did not differ systematically with regard to mean age at treatment start (11.6 vs. 11.3 years) or expected body weight% (78% vs. 80%). ARFID diagnoses were assigned retrospectively in a diagnostic re-evaluation after the introduction of DSM-5, using all available clinical information from the original treatment period together with follow-up information, as described in detail in [18]. Because this procedure also incorporated follow-up material, it is not possible to estimate the proportion of ARFID cases among non-participants with confidence.

### Measures

The primary variable of interest was the Swedish Eating Assessment for Autism Spectrum Disorders (SWEAA) total score. SWEAA is a self-report instrument assessing eating and mealtime problems in individuals with autism and normal intelligence [14]. The validation study showed good item-convergent and item-discriminant validity and differentiated between clinical and non-clinical groups. The total mean score cut-off is 12, with reported sensitivity of 0.70 and specificity of 0.50 [12]. SWEAA includes the following subscales: A, Perception; B, Motor control; C, Purchase of food; D, Eating behaviours; E, Mealtime surroundings; F, Social situation at mealtime; G, Other behaviour associated with disturbed eating; H, Hunger/satiety; and the single items I, Simultaneous capacity, and J, Pica.

SWEAA subscale K consists of five items derived from the Autism Spectrum Quotient (AQ) and covers broader autism-related features in social interaction, communication, behaviour, and attention to detail [3]. Because subscale K was not included in the original SWEAA validation and does not contribute to the SWEAA total score, it was treated as an exploratory measure rather than as a covariate in the main analyses.

Secondary measures were the Morgan-Russell Outcome Assessment Schedule (M-R OAS) [21] and the Eating Disorder Examination Questionnaire (EDE-Q) [27]. M-R OAS is a guided interview that assesses nutritional, menstrual, mental-state, psychosexual, and socioeconomic domains; the psychosexual domain was omitted here because of its heteronormative assumptions [15]. Total average scores range from 12 (excellent functioning) to 0 (poor functioning). Factor analysis has shown that the measure´s subscales all load onto one factor, accounting for one third of the variance. This property also points to the general relatedness of the subscales [21]. The EDE-Q includes 28 items and yields a global score plus the subscales restraint, eating concern, shape concern, and weight concern. Validation studies have found EDE-Q to have satisfactory concurrent validity [7], and internal consistency and temporal stability were deemed acceptable and test-retest reliability were found to be good [19].

Follow-up psychiatric diagnoses were established with a structured clinical interview [8], the structured clinical interview for DSM-IV-TR (SCID). SCID is a standardized diagnostic interview which in this study assesses the presence of mental disorders based on DSM-IV criteria. Validation studies have shown that SCID high inter-rater-reliability (kappa coefficients of at least 0.7) for major mental disorders, high diagnostic validity (as interview questions map directly onto DSM criteria), and high internal consistency (Cronbach´s alpha typically 0.8 or higher) [8]. For this study only the SCID I section was used, which covered acute, episodic or major mental disorders, but did not include autism. All SCID-interviews were made by an expert clinician with long experience in the eating disorders field (Riita Holmer) [18].

Height and weight were measured at follow-up. A stadiometer was used for stature measurement. Rounding was done to the nearest 0.1 centimeter. Weight measurement was done using a calibrated beam balance. Rounding was done to the nearest 0.1 kg. Weight was measured wearing underwear. Body mass index (BMI) was calculated as weight in kilograms divided by height in meters squared (kg/m^2^) [30].

In order to analyse outcome by different BMI-categories, three different BMI-categories were chosen: BMI < 20, BMI 20–25 and BMI > 25. These categories represent an approximation of underweight, normal and overweight categories. Although not adhering to strict WHO-definitions [29], here a cut-off at BMI 20 was chosen as the interval between BMI 18.5–20 is identified with risk for relapse in ED [10]. The BMI > 25 cut-off point corresponds to WHO-criteria for overweight in European populations, which is appropriate for the studied cohort [29]. As we also investigated BMI as a continuous independent variable in regard to SWEAA total score, our chosen BMI-categories levels should mainly be seen as an aid in visualizing autistic eating behaviour distribution in weight categories.

### Procedure

At follow-up, participants underwent structured diagnostic assessment, completed the SWEAA and EDE-Q, and were assessed with the M-R OAS. Height and weight were measured as part of the follow-up examination. Follow-up diagnostic status was then grouped into three categories: ongoing eating disorder, other psychiatric diagnosis without eating disorder, and no psychiatric diagnosis. Description of which non-ED diagnoses were identified in the sample is available in the article on the general psychiatric outcome [18].

### Statistical analysis

Statistical analyses were performed in SPSS. Because the distributions were non-normal, descriptive statistics are reported as medians, 25th and 75th percentiles, and ranges. Mann-Whitney U tests were used for comparisons between the AN and ARFID groups. Kruskal-Wallis tests were used to compare SWEAA, SWEAA-K, M-R OAS, and EDE-Q scores across follow-up diagnostic categories within the AN and ARFID groups and across BMI categories within each diagnostic group. For post-hoc analyses of significant results (*p* < 0.05), Dunn’s test with Bonferroni correction was used. Effect sizes for the Mann-Whitney U tests were calculated as Rosenthal’s r, with values of 0.1, 0.3, and 0.5 representing small, medium, and large effects, respectively [6]. Effect sizes for the Kruskal-Wallis test were calculated as eta-squared (η²) with values of 0.01, 0.06 and 0.14 representing small, medium and large effects, respectively [6]. A negative eta-squared value indicates negligible effect size. Because the subgroup analyses were exploratory and the sample was small results were interpreted cautiously and with attention to effect sizes. Linear regression was applied only in the ARFID group because the BMI-category results suggested an approximately monotonic pattern in ARFID, whereas the AN group showed a non-linear distribution of SWEAA scores across BMI categories.

## Results

### Follow-up diagnostic categories

Across all 56 participants, the median SWEAA total score was 9.53 (25th percentile 5.52, 75th percentile 13.9, range 0–40.6). Participants with an ongoing eating disorder at follow-up had the highest SWEAA scores (median 13.4), those with another psychiatric diagnosis had intermediate scores (median 9.94), and those with no diagnosis had the lowest scores (median 8.30). These differences were significant, *p* = 0.015, with a large effect size (η² = 0.12).

M-R OAS scores showed a similar graded pattern across follow-up diagnostic categories and differed significantly, *p* < 0.001, η² = 0.55.

### Comparison between baseline AN and ARFID groups

The overall median SWEAA total score was 10.0 in the AN group and 8.21 in the ARFID group. This difference was not statistically significant (*p* = 0.279). When the follow-up diagnostic subgroups were compared between AN and ARFID, the AN group tended to show somewhat higher symptom levels, but none of these between-group differences reached statistical significance. Thus, autistic eating behaviours appeared common in both diagnostic groups without clear evidence that they were more pronounced in AN than in ARFID.

### Within-group analyses by follow-up diagnostic status

Within both the AN and ARFID groups, the highest SWEAA total scores were seen in participants with an ongoing eating disorder, intermediate scores were seen in those with another psychiatric diagnosis, and the lowest scores were seen in those with no diagnosis (Table 1). The distribution of individuals scoring above the clinical cut-off for SWEAA-total score, per diagnostic follow-up status, are listed in Table 3. In the AN group, the Kruskal-Wallis tests showed significant differences for SWEAA total score (*p* = 0.038; post-hoc test (Dunn’s test): no diagnosis versus ED *p* = 0.034; no diagnosis versus other diagnosis *p* = 0.869; other diagnosis versus ED *p* = 0.574), and M-R OAS (*p* < 0.001; post-hoc test (Dunn’s test): no diagnosis versus ED *p* = 0.000; no diagnosis versus other diagnosis *p* = 0.005; other diagnosis versus ED *p* = 0.522), both with large effect sizes. In the ARFID group, M-R OAS differed significantly across follow-up diagnostic categories (Kruskal-Wallis test *p* = 0.019, post-hoc (Dunn’s test): other diagnosis versus no diagnosis *p* = 0.037; no diagnosis versus ED *p* = 0.103; other diagnosis versus ED *p* = 1.0), whereas SWEAA total score did not. SWEAA-K showed relatively little variation across diagnostic categories. EDE-Q scores did not show a clear or consistent pattern across the follow-up diagnostic groups.

### Within-group analyses by BMI category

BMI was examined in three descriptive categories: BMI < 20, BMI 20–25, and BMI > 25 (Table 2). In both the AN-group and the ARFID-group, the lowest SWEAA total median score was observed in the BMI < 20 category. In the AN-group, the BMI 20–25 category had an intermediate median score level and the BMI > 25 category had the highest median score. However, the BMI 20–25 category had a much larger score range than the BMI > 25 category, such that a linear relationship between BMI and SWEAA total score was deemed improbable. In the ARFID group the highest SWEAA scores were observed in the BMI > 25 category. M-R OAS scores broadly mirrored this pattern, with the highest scores in the low-BMI category and lower scores in the higher BMI categories. SWEAA-K scores were relatively evenly distributed across BMI groups. In contrast to the other variables, EDE-Q scores differed from the above patterns in the following manner: the AN-group had relatively low scores evenly distributed across the BMI-categories, whereas the ARFID-group had more elevated scores which also were evenly distributed across the BMI-categories. Neither the primary or any of the secondary outcome variables had significant differences when comparing the BMI-categories across the AN-group or ARFID-group. The distribution of individuals scoring above the SWEAA-total cut-off score for clinical group from the AN-group and ARFID-group are listed per BMI-group in Table 3.

Because the ARFID-group data suggested a monotonic association between BMI and SWEAA total score, a simple linear regression was performed in this group only. BMI significantly predicted SWEAA total score in the ARFID group according to the fitted model SWEAA total score = -17.75 + 1.28 × BMI, R² = 0.27, F(1,17) = 6.289, *p* = 0.023. The data met core assumptions for linearity, homoscedasticity and there were no extreme outliers.

**Table 1:** Outcome in the AN-group (*n* = 37) and ARFID-group (*n* = 19)

	AN-group			ARFID-group		
	On-going ED	Other Diagnosis (No ED)	No Diagnosis	On-going ED	Other Diagnosis (No ED)	No Diagnosis
	(*n* = 8)	(*n* = 9)	(*n* = 20)	(*n* = 5)	(*n* = 5)	(*n* = 9)
SWEAA total score median, (25th & 75th percentile, min-max)	14.28 (9.84, 31.8, 8.09–40.6) p(AN) = 0.038; η²(AN) = 0.133	10.1 (6.45, 18.3, 3.38–24.9)	8.77 (5.30, 11.7, 0.00-18.7)	12.6 (5.49, 29.0, 2.10–33.1) p(ARFID) = 0.284; η²(ARFID) = 0.032	8.21 (4.33, 13.7, 3.82–15.8)	7.16 (3.17, 10.1, 1.39-22.0)
SWEAA K	35.0 (26.5, 47.5, 20.0–65.0) p(AN) = 0.251; η²(AN) = 0.022	30.0 (17.5, 35.0, 15.0–40.0)	30.0 (20.0, 35.0, 0.00–45.0)	30.0 (22.5, 50.0, 20.0–55.0) p(ARFID) = 0.584; η²(ARFID)=-0.058	20.0 (15.0, 45.0, 15.0–45.0)	30.0 (10.0, 37.5, 0.00–45.0)
Morgan-Russell OAS average score	7.04 (3.69, 9.52, 2.25–9.67) p(AN) < 0.001; η²(AN) = 0.66	9.96 (8.81, 10.5, 7.04-11.0)	11.8 (10.9, 12.0, 8.91-12.0)	8.00 (6.10, 11.8, 4.88-12.0) p(ARFID) = 0.019; η²(ARFID)=-0.37	9.28 (8.71, 9.52, 8.63–9.58)	11.5 (10.7, 12.0, 10.2–12.0)
EDE-Q	0.475 (0.163, 2.84, 0.10–4.30) p(AN) = 0.617; η²(AN)=-0.03	0.800 (0.185, 2.25, 0.00-3.26)	0.410 (0.155, 0.940, 0.00-3.81)	2.92 (1.29, 3.12, 0.35–3.15) p(ARFID) = 0.112; η²(ARFID)=-0.15	1.07 (0.425, 2.28, 0.40–2.35)	1.25 (0.130, 1.80, 0.00-2.67)

SWEAA total score clinical cut-off = 12. p values and η² values in Table 1 refer to within-diagnosis comparisons across follow-up diagnostic categories (separately within AN and within ARFID)

**Table 2:** Outcome by BMI-category for AN- and ARFID-groups

	AN-group			ARFID-group		
	BMI < 20 (median = 19.3; range 16.5–19.9)	BMI 20–25	BMI > 25	BMI < 20 (median = 19.3; range 16.5–19.9)	BMI 20–25	BMI > 25
	(*n* = 12)	(*n* = 22)	(*n* = 3)	(*n* = 6)	(*n* = 9)	(*n* = 4)
SWEAA total score median, (25th & 75th percentile, min-max)	8.99 (5.53, 10.4, 0.00-29.1) p(AN) = 0.445; η²(AN)= -0.011	10.9 (6.73, 16.9, 1.06–40.6)	13.1 (8.30, 13.1, 8.30–13.4)	6.35 (1.92, 9.9, 1.39–10.5) p(ARFID) = 0.303; η²(ARFID) = 0.0244	8.21 (5.06, 14.2, 1.81-22.0)	16.3 (5.28, 31.1, 4.52–33.1)
SWEAA K	32.5 (21.3, 35.0, 0.00–40.0) p(AN) = 0.952; η²(AN)= -0.088	30.0 (20.0, 40.0, 15.0–65.0)	30.0 (25.0, -, 25–35)	25.0 (18.8, 36.3, 15.0–40.0) p(ARFID) = 0.646; η²(ARFID)= -0.070	30.0 (15.0, 45.0, 5.00–45.0)	40.0 (8.75, 52.5, 0.00–55.0)
Morgan-Russell OAS average score	11.9 (10.7, 12.0, 2.25-12.00) p(AN) = 0.056; η²(AN) = 0.111	10.5 (8.89, 11.3, 3.00–12.0)	9.67 (8.91, -, 3.0–12.0)	11.8 (8.47, 12.0, 8.00–12.0) p(ARFID) = 0.452; η²(ARFID)= -0.026	10.2 (9.37, 11.3, 8.80–12.0)	8.94 (5.49, 11.6, 4.88-12.0)
EDE-Q	0.43 (0.080, 0.838, 0.00-4.30) p(AN) = 0.726; η²(AN)= -0.040	0.555 (0.145, 2.28, 0.00-3.81)	0.450 (0.370, -, 0.37–2.20)	1.24 (0.045, 3.11, 0.00-3.15) p(ARFID) = 0.66; η²(ARFID)= -0.073	1.25 (0.375, 1.25, 0.20–2.67)	1.90 (0.768, 2.75, 0.50–2.92)

p values and η² values in Table 2 refer to within-diagnosis comparisons across BMI categories (separately within AN and within ARFID)

**Table 3:** Distribution of SWEAA-total median scores above cut-off for clinical group for AN-group and ARFID-group per diagnostic follow-up category and per BMI-group

	Eating disorder (*n*(%))	Other diagnosis (*n*(%))	No diagnosis (*n*(%))
AN-group	5 (62.5%)	4 (44.4%)	5 (25%)
ARFID-group	3 (60.0%)	1 (20%)	1 (11.1%)
	**BMI < 20**	**BMI 20–25**	**BMI > 25**
AN-group	2 (16.7%)	10 (45.5%)	2 (66.7%)
ARFID-group	0 (0%)	3 (33.3%)	2 (50.0%)

Cut-off score for clinical group: SWEAA-total score > 12

## Discussion

This long-term follow-up study examined autistic eating behaviours in former patients with childhood-onset AN and low-weight ARFID. Three findings were central. First, autistic eating behaviours were common in both groups at follow-up. Second, higher SWEAA scores were associated with concurrent psychopathology, particularly an ongoing eating disorder. Third, the lowest SWEAA scores were observed in the low-BMI category in both the AN-group and the ARFID-group, an unexpected pattern that merits cautious interpretation.

The overall similarity between the AN-group and the ARFID-group is notable. Previous literature has established an association between AN and autism, and more recent work has suggested a comparable overlap in ARFID [25, 28, 31]. Our findings are consistent with the view that autistic eating behaviours are not unique to one restrictive eating disorder presentation. Although SWEAA scores tended to be somewhat higher in the AN group, we found no statistically significant differences between the AN-group and the ARFID-group. Taken together, the present findings support the idea that autism-related eating characteristics may remain clinically relevant in both diagnostic groups many years after treatment.

The association between higher SWEAA scores and ongoing psychiatric morbidity at follow-up is clinically important. Participants with an ongoing eating disorder had the highest SWEAA scores, whereas those with no diagnosis had the lowest scores. This pattern suggests that autistic eating behaviours are linked to current clinical burden rather than demonstrating that SWEAA predicts long-term prognosis [23]. The similar gradient seen on the M-R OAS supports the internal coherence of the follow-up assessment and indicates that the diagnostic grouping captured meaningful differences in current functioning.

The low SWEAA scores in the BMI < 20 group were unexpected and should be interpreted cautiously. Several explanations are possible. First, BMI at long-term follow-up may not map directly onto autistic eating behaviours; SWEAA captures rigidity, sensory features, and mealtime social difficulties rather than weight status per se [14]. Second, the low-BMI category may have been clinically heterogeneous and could have included participants who remained constitutionally lean or who had residual low weight without the broader eating-related features measured by SWEAA [26]. Third, the subgroup sizes were small, and unstable estimates may therefore have influenced the observed pattern. The fact that the ARFID group showed a positive linear association between BMI and SWEAA score, whereas the AN group showed a more non-linear pattern, further suggests that the relationship between body weight and autistic eating behaviours is complex rather than straightforward.

SWEAA subscale K showed relatively little variation across the follow-up diagnostic categories. Because this subscale was not part of the original SWEAA validation and does not contribute to the SWEAA total score, these results should be considered exploratory. The absence of strong variation in SWEAA-K may indicate that broader autism-related features were relatively evenly distributed across the sample, whereas the SWEAA total score captured more clinically relevant variation in autism-related eating behaviours [14]. Caution is warranted given both the absence of psychometric validation of subscale K, and the low power in the present study.

EDE-Q, in contrast, did not clearly differentiate the groups. This may reflect not only the content of the instrument but also limitations in its underlying construct validity across specific subgroups. A factor-analytic study of the adolescent version of the EDE-Q, [9] found that the original four-factor structure was not supported and that, in girls, the questionnaire was largely characterized by a single general factor centred on dissatisfaction with shape and weight, while the structure in boys differed further [9]. These findings suggest that EDE-Q may primarily capture traditional weight- and shape-based eating-disorder psychopathology rather than the more sensory, rigid, socially mediated, or autism-related eating features assessed by SWEAA. In the present long-term follow-up sample, which included participants with ARFID and heterogeneous current diagnostic status, such a construct emphasis may have reduced the ability of EDE-Q to distinguish clinically relevant differences between groups. Lack of statistical power in this study may also explain the lack of significant differences in the EDE-Q results.

These findings may have practical implications. In long-term follow-up of both AN and ARFID, clinicians may benefit from assessing autistic eating behaviours explicitly rather than assuming that autism-related features are relevant only in acute AN or only in ARFID. Elevated SWEAA scores may help identify individuals with ongoing psychiatric burden who could need more individualized follow-up and support, including interventions that account for sensory sensitivities, rigid eating routines, and social difficulties around meals. Simultaneously, the findings also indicate that a person within a low weight-range, may not have exacerbated autistic eating behaviours or ED symptoms. It is worth noting that in the original validation study, a non-clinical group of neurotypical individuals had a mean SWEAA score of 3.8 [14], which provides context for the scores observed in our sample.

Several limitations should be acknowledged. SWEAA was the primary measure and provides a proxy for autistic eating behaviours rather than a direct autism diagnosis. The true rate of autism in the sample is therefore unknown. Some conceptual overlap may also exist between autism-related eating features and characteristics of restrictive eating disorders. The sample was small and selected, with 55% participation at follow-up, and comprised early-onset, low-weight cases, which limits generalizability. ARFID diagnoses were assigned retrospectively, which introduces diagnostic uncertainty. BMI is also an imperfect and sometimes controversial indicator that may not fully capture nutritional or clinical status. Strengths of the study include the long follow-up interval, the use of a validated Swedish instrument for autistic eating behaviours, and the inclusion of a structured diagnostic interview at follow-up, which strengthens the consistency of diagnostic classification.

Future research should examine these issues in larger prospective samples with repeated measurement of autistic eating behaviours and direct assessment of autism. Replication is especially needed for the BMI-related findings and for the question of whether autism-related eating characteristics differ meaningfully between AN and ARFID once broader autistic traits are measured directly.

## Conclusions

Autistic eating behaviours were common at long-term follow-up in both childhood-onset AN and low-weight ARFID. Higher SWEAA scores were associated with concurrent psychopathology, particularly an ongoing eating disorder, but autistic eating behaviours were not more pronounced in AN than in ARFID. The unexpectedly low SWEAA scores in the low-BMI group require replication and further explanation. Overall, the study adds to the evidence that autism-related eating characteristics are clinically relevant in both AN and ARFID.

## Data Availability

Data may be made available on reasonable request to corresponding author.
